# Investigation of urinary miRNA profile changes in amphotericin B-induced nephrotoxicity in C57BL/6 mouse, Sprague–Dawley rats and Beagle dogs

**DOI:** 10.1093/toxsci/kfaf029

**Published:** 2025-02-27

**Authors:** Adeyemi O Adedeji, Michael R Tackett, Genesis Tejada, James E McDuffie

**Affiliations:** Genentech, A Member of the Roche Group, South San Francisco, CA 94080, United States; Abcam, Waltham, MA 02453, United States; Abcam, Waltham, MA 02453, United States; Johnson & Johnson Innovative Medicine, La Jolla, CA 92121, United States

**Keywords:** biomarkers, urinary, miRNA, amphotericin B, nephrotoxicity

## Abstract

MicroRNA (miRNAs) have been associated with drug-induced kidney injury (DIKI). However, there are few reports on the utility of miRNAs, when monitoring for nephrotoxicity across multiple species. The purpose of this study was to assess the value of urinary miRNA profile changes as renal safety biomarkers, when monitoring for kidney injury in investigative toxicology studies. To this end, we evaluated urine miRNA expression levels in response to amphotericin B (AmpB)-induced nephrotoxicity in mice, rats, and dogs. The results showed that 35 miRNAs were significantly differentially expressed across the 3 species in response to the induced renal injuries. Dogs showed the highest number of miRNAs with significant changes. miR-205-5p and miR-31-5p were the most consistently altered miRNA biomarkers across all 3 species. In rodents, these 2 miRNAs were the most sensitive markers and showed comparable or better sensitivities than the previously published urine protein biomarkers with the same nephrotoxicant. In dogs, none of the upregulated miRNAs were as sensitive as urine clusterin protein as observed in a previously published study with AmpB. Taken together, these miRNAs could complement the more established urinary protein biomarkers in monitoring DIKI in mice, rats, and dogs. To our knowledge, this is the first report that demonstrates the comparative utility of urinary miRNAs for the early detection of DIKI across 3 nonclinical animal models.

Drug-induced kidney injury (DIKI) is commonly identified during drug development and serves as a dose-limiting factor or cause of attrition for several therapeutics. Serum creatinine (sCr) and blood urea nitrogen (BUN) are the standard biomarkers for detecting renal injury. However, these biomarkers do not significantly change until two-thirds to three-fourths of the nephrons are nonfunctional (Tripathi and Gregory 2015). As such, several novel urinary biomarkers with better sensitivity than sCr and BUN have been identified in preclinical animal models for the early detection of drug-induced nephrotoxicity (Bonventre et al. 2010; Dieterle et al. 2010a, 2010b; Ozer et al. 2010; Sistare et al. 2010; Vaidya et al. 2010; Vlasakova et al. 2014; Adedeji et al. 2019).

MicroRNAs (miRNAs or miRs) are small noncoding RNAs that regulate gene expression by degrading or suppressing the translation of their target messenger RNAs (mRNAs) (Benfey 2003; Chung and Lan 2015; Leti et al. 2015; Mai et al. 2015; Pandit and Milosevic 2015). Identified in 1993, miRNAs bind, destabilize, cleave, repress, and decay of target mRNAs via unique mechanisms which may prevent the production of proteins that play critical roles in almost all biological processes including cell proliferation, differentiation and development, apoptosis, metabolism, immunity, oncogenesis, and viral replication (Lee et al. 1993). Kidney-specific or kidney-enriched miRNAs have been previously discovered (Sun et al. 2004; Naraba and Iwai 2005), with subsequent studies showing the putative roles of miRNAs in select kidney functions (Kato et al. 2007; Harvey et al. 2008; Ho et al. 2008; Shi et al. 2008; Wang et al. 2008). To date, only a fraction of these kidney-enriched miRNAs has been correlated to renal pathology (Chorley et al. 2021); and the functions and targets are known for even fewer of these miRNAs (Huang et al. 2019). Differentially expressed miRNAs in response to injuries have been identified in different regions of the nephron in rats (Chorley et al. 2021). For example, miR-23a-3p was identified as a marker specific to glomerular injury, and miR-192-5p was responsive to proximal tubular injury, miRs-221-3p, -210-3p, -222-3p, and -17-1-3p demonstrated low to medium expression and, depending on the platform, displayed a distal expression pattern (thick ascending loop of Henle and/or collecting ducts). More recently, a panel of 6 miRNAs (miRs-210-3p, 423-5p, 143-3p, 130b-3p, 486-5p, 193a-3p) was revealed following analysis of urine sampled across multiple time points from cynomolgus monkeys exposed to a nephrotoxic antibody drug conjugate (Dasgupta et al. 2024). The results showed that exosome-associated miRNA expression level changes showed distinct trends relative to miRNAs quantified in whole urine, indicating a different urinary excretion mechanism of miRNAs than those released passively into the urine.

miRNAs have been measured from tissues and biofluids using several assays including quantitative real-time PCR (qRT-PCR), digital PCR, microarray, and high-throughput small RNA-sequencing. These methods require RNA isolation, and in some instances small RNA enrichment, and/or reverse transcription before proceeding to miRNA quantification (Precazzini et al. 2021). Here, we report the results of a study conducted by the Predictive Safety Consortium-Nephrotoxicity Working Group (PSTC-NWG)—a working group of experienced biopharmaceutical industry pathologists, toxicologists, and life scientists who are collaboratively involved in developing novel renal biomarkers for regulatory considerations for nonclinical and clinical drug development applications. We assessed the response of miRNA biomarkers to Amphotericin B (AmpB)-induced kidney injury in C57BL/6 mice, Sprague–Dawley rats, and beagle dogs using a combination of predesigned and custom FirePlex miRNA assays for the direct multiplex profiling of up to 400 miRNAs in urine samples, without the need for RNA isolation. We identified 35 miRNAs that were significantly differentially expressed across these species in response to the AmpB-induced renal injuries. These results add to the body of knowledge of renal safety biomarkers by providing miRNA panels that could complement the more established urinary protein biomarkers used for monitoring DIKI in nonclinical toxicity studies.

## Materials and methods

### Test article and vehicle

Test article, Amphotericin B USP grade powder (X-Gen Pharmaceuticals, Inc, Horseheads, NY, United States) was formulated in the vehicle, 5% (w/v) dextrose solution for injection USP grade (Baxter, Deerfield, IL, United States or Hospira, Inc., Lake Forest, IL, United States).

### Ethical statement

All in vivo animal procedures were conducted in an Association for Assessment and Accreditation of Laboratory Animal Care International (AAALAC) accredited facility under an Institutional Animal Care and Use Committee approved protocol at Johnson & Johnson Innovative Medicine, San Diego, CA, United States. Except when noted, standard procedures and conditions for animal care, housing, access to water and food, environment, and room maintenance were used. All other procedures were performed in accordance with laboratory standard operating procedures or established laboratory best practices. Moreover, these studies were conducted in accordance with the 3Rs (Replacement, Reduction, and Refinement) policy.

### C57BL/6 mouse AmpB study

#### Animals, test article administration, biofluid collections, histopathology, and urinary miRNA measurements

**Fig. 1.**
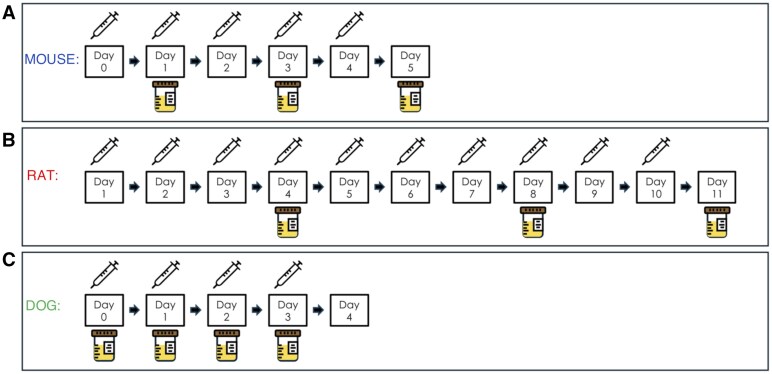
AmpB administration and urine collection schemes. A) Mice were administered via lateral tail vein, a slow intravenous (IV) infusion (1 ml/min) of either a single dose of AmpB (2 mg/kg/d) or vehicle (5% dextrose) on study days 1 to 4, and representative urine samples were collected on study days 1, 3, and 5 postdose. B) Rats received bolus intravenous injections of either AmpB (3 mg/kg/dose) or Vehicle (5% w/v dextrose solution) at 2 ml/kg/dose via lateral tail vein for 10 consecutive days and representative urine samples were collected on study days 4, 8, and 11 postdose. C) Dogs were intravenously dosed once daily for 4 consecutive days with 0.3 mg/kg of AmpB or vehicle (5% dextrose) and representative urine samples were collected on study days 0 (predose on day −5), 1, 2, and 4 postdose.

The information about the animals, test article administration, biofluid collections, and histopathology have been previously published (Adedeji et al. 2021). Briefly, approximately 7-wk-old male C57BL/6 mice purchased from Charles River Laboratories (Hollister, CA, United States) were randomly assigned to either a control group or a test article group (4 per group). There were 3 control, and 3 test article groups assigned for terminal sample collection and necropsy on either day (D) 1, 3, or 5 of the study. On studies D1 to D4, mice were administered via lateral tail vein, a slow intravenous (IV) infusion (1 ml/min) of either a single dose of AmpB (2 mg/kg/d) or vehicle (5% dextrose) (Fig. 1A). The dose and duration of dosing were selected based on the results of an initial pilot dose range finding study conducted in mice to identify the minimal toxic effect on kidneys and No Observable Effect Level (NOEL) for AmpB-induced changes in serum chemistry (e.g. sCr, BUN) parameters and kidney histopathology (data not shown). For the D1 animals, urine samples were collected for 8 h on wet ice after dosing, whereas urine samples were collected overnight (∼16 h) on wet ice from the D3 and D5 animals. Urine samples aliquoted for downstream biomarker analysis were centrifuged at 2,900 rpm at 4 °C for 10 min and stored at 80 °C until time of analysis. Urine miRNAs, protein biomarkers, and creatinine were all measured within 30-d postfinal necropsy, and all biomarkers were normalized with individual urine creatinine concentrations.

One control group and 1 test article group were euthanized on D1, D3, or D5 of the study by CO_2_ inhalation and exsanguination via vena cava blood collection into serum separation tubes. Following euthanasia, the kidneys were fixed in 10% neutral-buffered formalin and embedded in paraffin wax, sectioned at 4 μm, stained with hematoxylin and eosin (H&E), and examined microscopically by the study pathologist. Injury severity grades were designated using a 0 to 5 grading scale: 0 (no observable pathology), 1 (minimal), 2 (mild), 3 (moderate), 4 (marked), and 5 (severe). Mouse urinary miRNAs were measured using the FirePlex Rodent Discovery Panel that is well described in detail in the “FirePlex MiRNA Assay” section below. Vehicle-treated mouse urine samples were utilized as concurrent control samples for the study.

### Sprague–Dawley rat AmpB study

#### Animals, test article administration, biofluid collections, histopathology, and urinary miRNA measurements

The detailed information about the animals, test article administration, biofluid collections, and histopathology have been previously published (McDuffie et al. 2016b). Briefly, 6-wk-old naïve female Sprague–Dawley rats [Crl: CD(SD)] purchased from Charles River Laboratories (Hollister, CA, United States) were randomly assigned to either a control group or test article group (8/group). Rats received bolus intravenous injections of either AmpB (3 mg/kg/dose) or Vehicle (5% w/v dextrose solution) at 2 ml/kg/dose via a lateral tail vein for 10 consecutive days (Fig. 1B). The dose and the frequency of dosing were based on the methods described in the literature (LeBrun et al. 1996, 1999) and an initial pilot dose range finding study conducted in rats to identify the minimal toxic effect on kidneys and NOEL for AmpB-induced changes in serum chemistry (e.g. sCr, BUN) parameters and kidney histopathology (data not shown). Serial urine samples were collected from animals that were fasted. For urine collections, the animals were individually housed in metabolism cages. Urine samples were collected on ice, overnight during an 18-h collection period (between 3 PM and 9 AM). On D4, D8, and D11, total urine volumes (output) were recorded. Urine samples were centrifuged at 3,000×*g* at 4 °C for 10 min and stored at −80 °C until the time of analysis. Urine miRNAs, protein biomarkers, and creatinine were all measured within 30-d postfinal necropsy, and all biomarkers were normalized with individual urine creatinine concentrations. On D11 (the day of scheduled study termination), all animals were euthanized by CO_2_ inhalation followed by exsanguination. Representative kidney (left and right) tissues were collected from the animals and were fixed in 10% neutral-buffered formalin for at least 72 h. Preserved tissues were then trimmed, embedded in paraffin, sectioned at 5 μm, mounted on glass slides, and stained with H&E. Microscopic examination of the H&E-stained slides was performed by the study pathologist and the kidney lesion severity grading scores were characterized as minimal, mild (slight), moderate, marked, and severe.

Rat urinary miRNAs were measured using the FirePlex Rodent Discovery Panel that is well described in detail in the “FirePlex MiRNA Assay” section below. Vehicle-treated rat urine samples were utilized as concurrent control samples for the study.

### Beagle dog AmpB study

#### Animals, test article administration, biofluid collections, histopathology, and urinary miRNA measurements

The information about the animals, test article administration, biofluid collections, and histopathology have been previously published (Adedeji et al. 2023). Briefly, nonnaïve male and female beagle dogs purchased from Marshall Farms, North Rose, NY, United States, were individually housed. Animals were randomly designated to either a control group or a test article group (6 per group; 3 males and 3 females in each group). Dogs were intravenously dosed once daily for 4 consecutive days with 0.3 mg/kg of AmpB (X-Gen Pharmaceuticals, Inc., Big Flats, NY, United States) which was formulated in vehicle (5% dextrose solution for injection, USP, Hospira, Inc., Lake Forest, IL, United States) (Fig. 1C). The dose, route of administration, and duration of dosing were selected based on literature (Keim et al. 1976; Gerkens et al. 1983; Rubin et al. 1989; Brooks et al. 1991) and initial pilot dose range finding study conducted in dogs to identify the minimal toxic effect on kidneys and NOEL for AmpB-induced changes in serum chemistry (e.g. sCr, BUN) parameters and kidney histopathology (data not shown). Using a pan catch method, representative urine samples were collected on wet ice for an 18-h period overnight predose (on day 5) and after daily dosing. Urine collected overnight on ice after the first dose (but before the second dose was administered) is referred to as D1. Similarly, urine overnight collection after second and fourth doses have been referred to as D2 and D4, respectively. Total urine volumes were recorded and immediately placed on wet ice for up to 1 h prior to centrifugation at 800×*g* for at least 10 min at 4 °C. Urine supernatants were prepared as aliquots and stored at −80 °C until the time of analysis. Urine miRNAs, protein biomarkers, and creatinine were all measured within 30-d postfinal necropsy, and all biomarkers were normalized with individual urine creatinine concentrations. After 4 d of dosing, animals were euthanized, and representative kidney tissues were collected and fixed in 10% neutral-buffered formalin. The formalin-fixed kidney (left and right) samples were processed, embedded in paraffin, sectioned at 4 μm, mounted on glass slides, deparaffinized, and stained with H&E for subsequent microscopic evaluation in a blinded fashion by a pathologist. Severity grading was assigned based on the classification of microscopic evidence of lesions which was characterized as minimal, mild (slight), moderate, marked, or severe.

Dog urinary miRNAs were measured using a customized FirePlex miRNA Panel described in detail in the “FirePlex MiRNA Assay” section below. Vehicle-treated dog urine samples were utilized as concurrent control samples for the study.

### FirePlex MiRNA assay

For mouse and rat urine samples, the FirePlex Rodent Discovery Panel (ab217052, Abcam, Waltham, MA, United States) consisting of 6 panels profiling a total of 400 unique miRNAs was used. The FirePlex technology workflow has been previously published (Tackett and Diwan 2017). The technology utilized hydrogel particles and conventional flow cytometry, enabling multiplex capture of miRNAs with picomolar sensitivity and high specificity. No prevalidated canine panels were available, so to profile the dog urine samples, customized sets of 2 panels were created profiling a total of 120 unique miRNAs. The contents of the customized canine panels were chosen based on a literature review of miRNAs in kidney toxicology (Ichii et al. 2014). A complete list of all targets profiled for each species is reported in Tables S1 and S2. All supplementary information can be accessed at https://doi.org/10.5061/dryad.c59zw3rk4. The contents of the rodent panels were validated for use in rodent plasma and sera but not in urine, and the contents of the canine panels were not validated prior to use.

Briefly, urine (100 μl for rat and mouse samples and 40 μl for dog samples) was mixed with an equal volume of Lysis Mix and incubated at 60 °C for 45 min with shaking. For each panel, 35 μl of FirePlex Particles were added to a well of a 96-well filter plate and filtered. Twenty-five microliters of lysate and 25 μl of hybridization buffer were mixed and incubated at 37 °C for 60 min while shaking. After 2 rinses, 75 μl of 1× Labeling Buffer was added to each well and incubated at room temperature for 60 min while shaking. After 2 more rinses, samples were eluted with 65 μl of 95 °C RNAse-free water. Then, 30 μl of this eluant was mixed with 20 μl qRT-PCR master mix followed by 32 cycles of qRT-PCR amplification. Sixty microliters of hybridization buffer and 20 μl of the qRT-PCR product were added back to the particles in the corresponding well of the filter plate and were incubated at 37 °C for 30 min while shaking. After 3 rinses, 75 μl of Reporting Buffer was added to each well and incubated at room temperature for 15 min while shaking. After 2 final rinses, 175 μl of Run Buffer was added to each well and the plates scanned on an EMD Millipore Guava 8HT or 6HT flow cytometer. The flow cytometer output was analyzed with the FirePlex Analysis Workbench software. All samples were measured by assessing the mean fluorescent intensity (MFI) of the miRNAs in triplicates, and the uCr-normalized averages of the readouts were recorded.

### Data analysis

Raw data underwent 2 rounds of normalization prior to analysis. First, for each species, geometric average normalization was used to limit sample-to-sample assay variation across and between plates. These data were then further normalized against individual urine creatinine levels detected in the samples. The fraction of samples detecting a given miRNA above the calculated limit of detection is reported in Tables S1 (rodents) and S2 (dogs), whereas the raw data used for the analyses can be found in Tables S3 (mice), S4 (rats), and S5 (dogs). All supplementary information can be accessed at https://doi.org/10.5061/dryad.c59zw3rk4. We normalized each animal’s individual MFIs with the correlating individual urine creatinine concentration given the observation by Adedeji et al. (2019) that showed that urine creatinine was the best normalizing parameter for urinary biomarkers. The significance of differences of the urine creatinine-normalized averages of the MFI of the miRNAs between the AmpB-dosed group and the control group were then assessed by the Mann–Whitney *U*-test for pairwise comparison using Excel software for MacOS. Fold changes in biomarker concentration were expressed versus concurrent control values for each time point in each study. A 2-tailed *P*-value of <0.05 was considered to have statistical significance for all analyses.

Moreover, heatmaps were generated using the online tool Heatmapper. Hierarchical clustering was computed using the average linkage settings and Pearson was used to calculate the distance between rows and columns (Babicki et al. 2016).

## Results

### C57BL/6 mice: 5 miRNAs showed significant changes in response to AmpB-induced renal injury

**Fig. 2.**
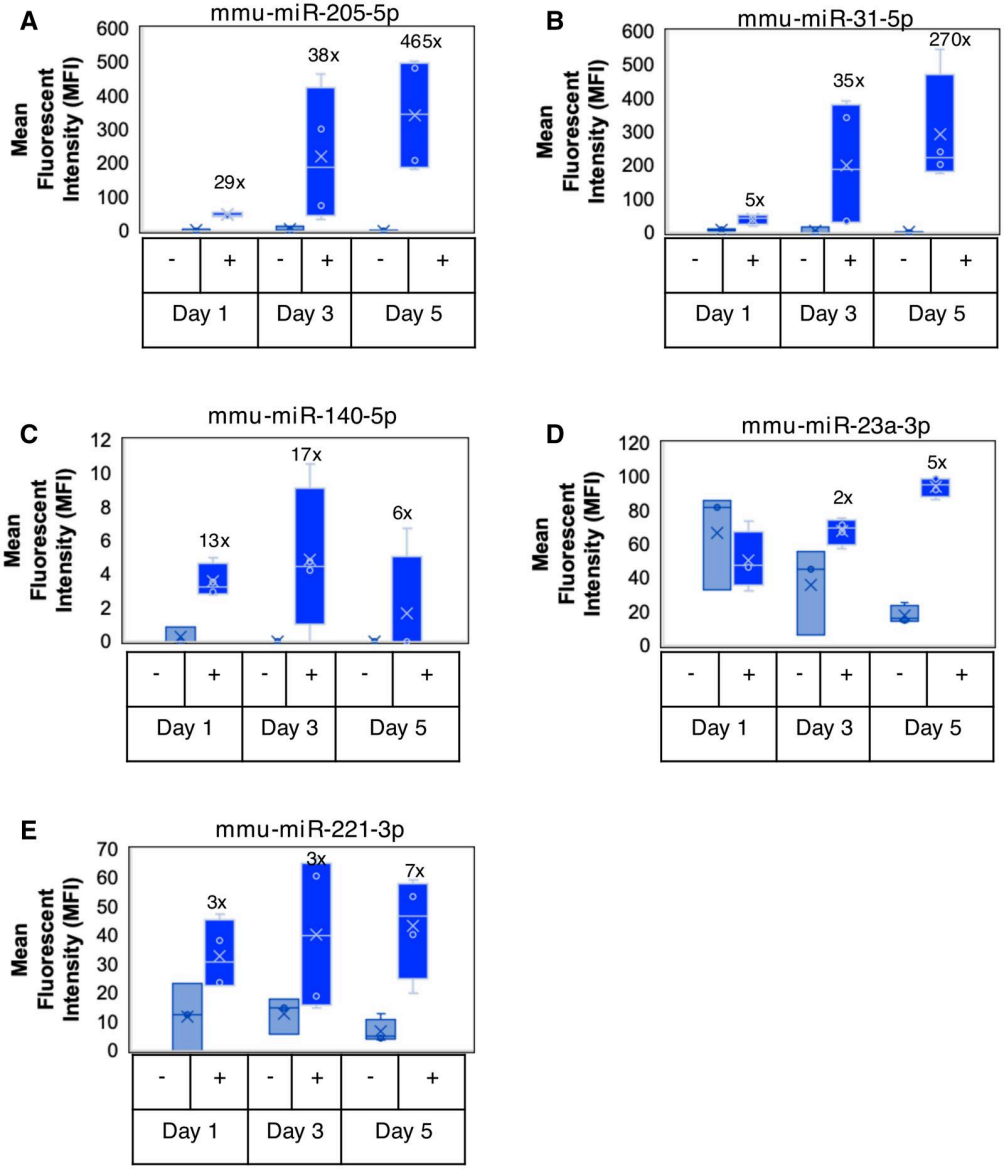
AmpB-induced changes in urinary miRs in male C57BL/6 mice. When compared with the concurrent controls, increases in MFI (after urine creatinine normalization) and fold change of urinary (A) mmu-miR-205-5p, (B) mmu-miR-31-5p, (C) mmu-miR-140-5p, (D) mmu-miR-23a-3p, and (E) mmu-miR-221-3p were observed over time. mmu, *Mus musculus*.

In a prior study (Adedeji et al. 2021), we showed that urinary albumin (ALB), kidney injury molecule 1 (KIM-1), neutrophil gelatinase-associated lipocalin (NGAL), and osteopontin (OPN) were significantly increased in response to AmpB-induced renal injury characterized by minimal to marked time-dependent renal tubular changes including renal tubular dilation, intraluminal granular and/or hyaline casts, epithelial cell necrosis, and interstitial inflammatory cell infiltration. Using the same urine samples, miRNAs were measured using the 400plex FirePlex platform as described in the methods. Of all the miRNAs measured, 5 miRNAs showed significant changes in response to the renal injury. These miRNAs included mmu-miR-205-5p (D1 to D5, ↑ 29× to 465×), mmu-miR-31-5p (D1 to D5, ↑ 5× to 270×), mmu-miR-140-5p (D1 to D5, ↑ 6× to 17×), mmu-miR-221-3p (D1 to D5, ↑ 2× to 7×), and mmu-miR-23a-3p (D3 to D5, ↑ 2× to 5×) (Fig. 2 and Table 1). Moreover, a heatmap showing the hierarchical clustering of the evaluated miRNAs, whose average expression was higher than the calculated limit of detection of the assay for all mice samples has been provided (Fig. S1 can be accessed at https://doi.org/10.5061/dryad.c59zw3rk4).

**Table 1.** Range of fold or percent changes of miRNAs across the animal species dosed with AmpB.

miRNA	Mouse	Rat	Dog
**miR-103**	NS	NS	4× to 7×^e^
**miR-107-3p**	NS	NS	4× to 7× ^e^
**miR-140-5p**	6× to 17×^&^ ^e^	NS	NS
**miR-148a**	NS	NS	3× to 21×^f^
**miR-151**	NS	NS	5× to 8×^e^
**miR-152**	NS	NS	5× to 9×^b^
**miR-15a-5p**	NS	NS	5× to 9×^b^
**miR-15b-5p**	NS	NS	4× to 9×^e^
**miR-16-5p**	NS	NS	3× to 5×^e^
**miR-191-5p**	NS	NS	4× to 9×^e^
**miR-194**	NS	NS	4× to 9×^b^
**miR-20b**	NS	NS	3× to 11×^b^
**miR-203-3p^$^**	NS	7× to 37×^e^	4× to 6×^b^
**miR-205-5p^#^**	29× to 465×^f^	67× to 164×^d^	2× to 6×^d^
**miR-221-3p^$^**	3× to 7× ^e^	NS	2× to 10×^d^
**miR-23a-3p^$^**	2× to 5×^c^	NS	3× to 7×^a^
**miR-23b-3p**	NS	NS	7× to 9×^b^
**miR-24-3p**	NS	NS	2× to 7×^b^
**miR-26a**	NS	NS	3× to 5×^b^
**miR-26b-5p**	NS	NS	5× to 15×^b^
**miR-27a-3p**	NS	NS	3× to 8×^a^
**miR-29b**	NS	NS	4× to 16×^e^
**miR-29c**	NS	NS	2× to 10×^d^
**miR-30a-5p**	NS	NS	4× to 5×^d^
**miR-30b-5p**	NS	NS	NM
**miR-30c-5p**	NS	NS	4× to 8×^f^
**miR-30d-5p**	NS	NS	3× to 5×^d^
**miR-31-5p^#^**	5× to 270×^a^	29× to 61×^b^	3× to 6×^b^
**miR-320a-3p**	NS	3× to 5×^d^	NS
**miR-328-3p**	NS	4× to 9×^e^	NS
**miR-375-3p**	NS	75% to 91%^b^ ↓	NS
**miR-423-5p**	NS	5× to 120×^a^	NS
**miR-429**	NS	4× to 5×^a^	4×^d^
**miR-652-3p**	NS	4× to 8×^b^	NS
**miR-660-5p**	NM	NM	4× to 10×^d^
**miR-6976-3p**	NS	NS	NM
**miR-93-5p**	NS	NS	4× to 5×^f^

NS, not significant; NM, not measured; #, changed in 3 species; $, changed in 2 species; &, relative to D1 concurrent control mean value.

a *P*-value <0.05.

b *P*-value <0.05 to <0.01.

c *P*-value <0.01.

d *P*-value <0.05 to <0.001.

e *P*-value <0.01 to <0.001.

f *P*-value <0.001.

### Sprague–Dawley rats: 9 miRNAs showed significant changes in response to AmpB-induced renal injury

**Fig. 3.**
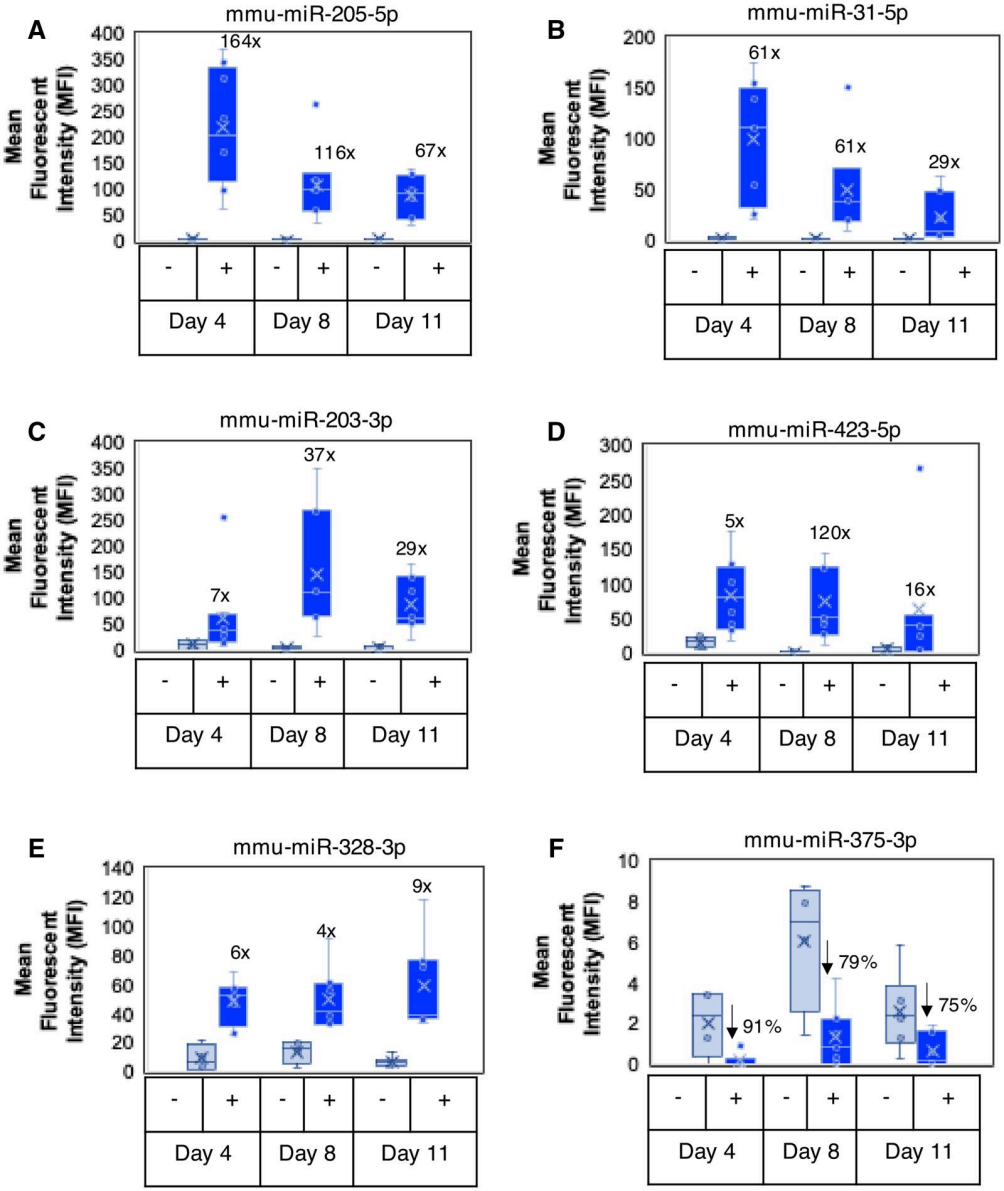
AmpB-induced changes in urinary miRs in female Sprague–Dawley rats. When compared with the concurrent controls, changes in MFI (after urine creatinine normalization) and fold/percent of urinary (A) mmu-miR-205-5p, (B) mmu-miR-31-5p, (C) mmu-miR-203-3p, (D) mmu-miR-423-5p, (E) mmu-miR-328-3p, and (F) mmu-miR-375-3p were observed over time. mmu, *Mus musculus*.

In a previously published study (McDuffie et al. 2016b), it was shown that urinary clusterin (CLU), ALB, KIM-1, NGAL, Cystatin C, total protein (TP), and OPN were significantly elevated in response to AmpB-induced renal injury characterized by slight to moderate multifocal lesions in histopathology. These included minimal to mild multifocal cortical tubular dilatation, basophilia, mineralization, intratubular granular eosinophilic casts along with acute pelvic inflammation. Slight to moderate medullary interstitial inflammation and intratubular inflammatory cell casts were also observed. Using the same urine samples for this study, 9 miRNAs showed significant changes in response to the renal injury. Significant increases were observed with mmu-miR-205-5p (D4 to D11, ↑ 67× to 164×), mmu-miR-31-5p (D4 to D11, ↑ 29× to 61×), mmu-miR-203-3p (D4 to D11, ↑ 7× to 37×), mmu-miR-423-5p (D4 to D11, ↑ 5× to 120×), mmu-miR-328-3p (D4 to D11, ↑ 4× to 9×), mmu-miR-320a-3p (D4 to D11, ↑ 3× to 5×), mmu-miR-429 (D4 to D11, ↑ 4× to 5×), and mmu-miR-652-3p (D4 to D11, ↑ 4× to 8×) (Fig. 3A–E and Table 1). In contrast, miR-375-3p was the only miRNA that showed decreases in response to renal injury (↓75% to 91%) between D4 and D11 of dosing (Fig. 3F and Table 1). Moreover, a heatmap showing the hierarchical clustering of the evaluated miRNAs, whose average expression was higher than the calculated limit of detection of the assay for all rat samples has been provided (Fig. S2 can be accessed at https://doi.org/10.5061/dryad.c59zw3rk4).

### Beagle dogs: 29 miRNAs showed significant changes in response to AmpB-induced renal injury

**Fig. 4.**
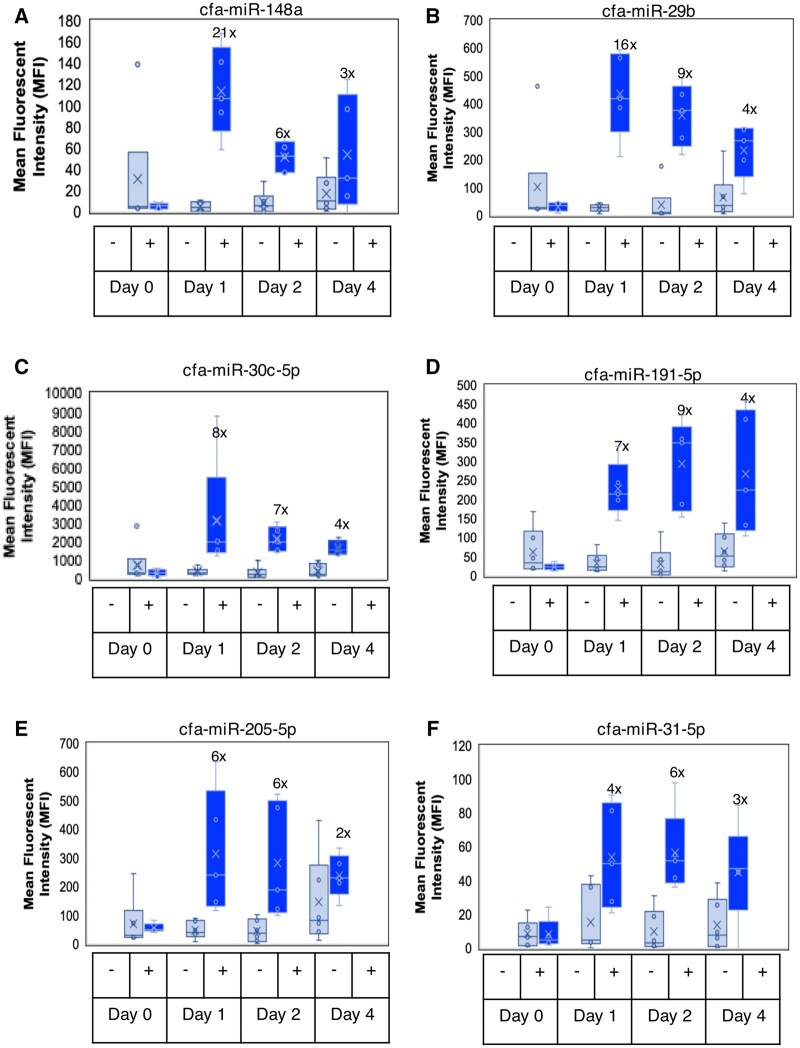
AmpB-induced changes in urinary miRs in male and female beagle dogs. When compared with the concurrent controls, increases in MFI (after urine creatinine normalization) and fold changes of urinary (A) cfa-miR-148a, (B) cfa-miR-29b, (C) cfa-miR-30c5p, (D) cfa-miR-191-5p, (E) cfa-miR-205-5p, and (F) cfa-miR-31-5p over time in beagle dogs dosed with Amphotericin-B. cfa, *Canis familiaris*.

Like the mouse and rat studies, we have previously published a report on the utility of renal biomarkers in response to AmpB-induced renal injury in beagle dogs (Adedeji et al. 2023). We showed that urine CLU was the most sensitive renal protein biomarker to renal tubular injury in dogs. In histopathology, the renal injury was characterized by minimal to mild multifocal treatment-related microscopic lesions in the kidneys characterized by tubular cell necrosis, degeneration, regeneration, dilatation, and mineralization. Additional treatment-related microscopic lesions in the kidney included hemorrhage and acute inflammation in the interstitium and intratubular granular casts. Using the same urine samples, the results showed that 29 miRNAs showed significant changes in response to the renal injury. The miRNAs with most significant changes considering the magnitude of change, relatively higher MFIs and lower *P*-values included cfa-miR-148a (D1 to D4, ↑ 3× to 21×), cfa-miR-29b (D1 to D4, ↑ 4× to 16×), cfa-miR-30c-5p (D1 to D4 ↑ 4× to 8×), and cfa-miR-191-5p (D1 to D4 ↑ 4× to 9×) (Fig. 4A–D and Table 1). Interestingly, as observed with the rat and mouse data, cfa-miR-205-5p (D1 to D4, ↑ 2× to 6×) and cfa-miR-31-5p (D1 to D4, ↑ 3× to 6×) also showed increases, albeit with a lower magnitude of change relative to what were observed with rat and mouse. Other noteworthy miRNAs with significant changes are noted in the dog section of Table 1. Moreover, a heatmap showing the hierarchical clustering of the evaluated miRNAs, whose average expression was higher than the calculated limit of detection of the assay for all dog samples has been provided (Fig. S3 can be accessed at https://doi.org/10.5061/dryad.c59zw3rk4).

### Six miRNAs showed consistency across 2 or more species

As shown in Table 1, 35 miRNAs were significantly differentially expressed across all species in response to the renal injuries. However, there were only 6 miRNAs that were consistently upregulated in response to renal injuries in 2 or more animal species, 2 of which were consistently upregulated in all 3 species evaluated. These included miR-205-5p and miR-31-5p that showed significant increases in mice (Fig. 2A and B), rats (Fig. 3A and B), and dogs (Fig. 4E and F); miR-221-3p and miR-23a-3p showed significant increases in mice (Fig. 2D; Table 1) and dogs (Table 1); and miR-203-3p and miR-429 that showed significant increases in rats (Fig. 3C; Table 1) and dogs (Table 1).

## Discussion

AmpB is an antifungal agent, but its use is often limited by nephrotoxicity (Noor and Preuss 2024). The nephrotoxicity of AmpB is attributed to several mechanisms including but not limited to direct renal tubular toxicity as it preferentially binds to ergosterol (a key component of fungal cell membranes) or cholesterol in renal tubular cells, leading to the formation of pores that disrupt cellular integrity. The resulting disruption of the tubular epithelial cells and eventual death and/or sloughing represents the origin of miRNAs in the urine (Hamill 2013). Moreover, AmpB is known to primarily affect the distal nephron, causing distal renal tubular acidosis by disrupting the normal function of the collecting duct cells, leading to impaired hydrogen ion secretion and subsequent potassium wasting in the urine, resulting in electrolyte imbalances like hypokalemia and metabolic acidosis (McCurdy et al. 1968; Da Silva Lugo 2020).

Here, we have been able to identify 35 miRNAs that were significantly differentially expressed across all species in response to the AmpB-induced renal injuries in this study. Six of these miRNAs showed significant changes in 2 or more species, 2 of which were consistently upregulated in all 3 species. One of the most important observations in this study was the interspecies upregulation of miR-205-5p and miR-31-5p in response to AmpB-induced acute kidney injury in mice, rats, and beagle dogs.

miR-205-5p is a highly conserved miRNA expressed in epithelial tissues of different species (Lim et al. 2003; Ryan et al. 2006). It has been widely characterized for its functions in normal development and in cancer. It is known to be highly expressed in human epithelial tissues of breast, prostate, skin, eye, and thymus, where it plays a crucial role in tissue morphogenesis and homeostasis. Generally, it sustains the epithelial phenotype through the direct targeting of zinc finger E-box-binding homeobox 1 (*ZEB1*) and *ZEB2*, 2 transcription factors that repress *E-cadherin* and other polarity genes (Gregory et al. 2008). In cancers, it has been reported to be abnormally expressed (either down- or upregulated) and have pro- or antitumorigenic roles depending on the cellular context and target genes. In the kidney, miR-205 is believed to play a protective and adaptive role, by attenuating renal injuries through renal cell apoptosis inhibition in hypoxia-induced renal injury (Chen et al. 2019), and by helping to defend critical epithelial layers of the urothelium and collecting duct from the mechanical stress and physiological consequences of renal distention and urine stasis, as well as promoting cellular regeneration (Dhabhar et al. 1994). Moreover, a series of miRNAs including miR-205 was found enriched in the collecting duct by sRNA-seq and was significantly increased in urine from male Sprague–Dawley rats treated with a known collecting duct toxicant, compound Z (Chorley et al. 2021). Hence, these details are consistent with the upregulation of miR-205-5p in these AmpB-induced nephrotoxicity studies in rats, mice, and dogs.

miR-31-5p is also a highly conserved miRNA that is involved in diverse biological processes, including cell differentiation, proliferation, migration, and apoptosis in various tissues and organs, thereby affecting fertility, embryonic development, bone formation, vascular development, and myogenesis (Aboobaker et al. 2005; Leaman et al. 2005; Rouas et al. 2009; Cacchiarelli et al. 2011; Crist et al. 2012; Itkin et al. 2012; Baglio et al. 2013; Mizoguchi et al. 2013; Wang et al. 2013; Kresowik et al. 2014; Xie et al. 2014; Munoz et al. 2015; Stepicheva and Song 2016; Weilner et al. 2016). miR-31-5p has also been shown to be dysregulated in various diseases including cancer (Kim et al. 2015; Hou et al. 2016; Kent et al. 2016) and autoimmune diseases (Dhamne et al. 2013; Yadav et al. 2013). Several studies have shown that the roles of miR-31 are context-dependent as this complexity may be caused by its broad spectrum of molecular targets and specific expression in various tissues and organs (Stepicheva and Song 2016). In patients with lupus nephritis, upregulation of miR-31-5p has been observed and thought to inhibit HIF-1α thereby ameliorating the nephritis (Garcia-Vives et al. 2020). Given the established roles of miR-205-5p and miR-31-5p in kidney injury, we believe their responses to the nephrotoxic-induced acute kidney injury observed in preclinical animal species in this study would also be translatable to humans as sensitive acute kidney injury biomarkers for safety monitoring during drug development.

In a prior study, Chorley et al. (2021) showed the differential expressions of some miRNAs in both kidney tissues and urine samples of rats treated with nephrotoxicants that caused injuries to distinct nephron segments including the glomerulus, proximal tubule, thick ascending limb of the loop of Henle (TAL), and the collecting duct. They showed that miR-23a-3p was a marker specific for drug-induced glomerular injury, whereas miRs-140-5p and -192-5p were specific markers for proximal tubular injury. miRs-221-3p, -222-3p, and -210-3p were more specific for TAL injury and miRs-31a-5p, -205-5p, and -423-3p were considered specific for collecting duct injury. Some of these observations aligned with the changes noted in this study. As previously mentioned, AmpB primarily causes injury at the distal nephron and the consistent upregulation of miR-31a-5p and miR-205-5p in all species evaluated in this study, along with the upregulation of miR-221-3p in mice and dogs further confirmed the distal nephron-specificity of these miRNAs. That said, upregulations of miR-140-5p in mice, and miR-23a-3p in mice and dogs were suggestive of 2 possibilities: A), the possibility that these miRNAs could be expressed at the distal nephron of mice and dogs *vs* proximal nephron in rats; or b) that AmpB can exert injuries beyond the distal nephron. In mice and rats, the second possibility was supported by the fact that urinary ALB and KIM-1, 2 markers known to be specific for injuries to the proximal nephron (Bonventre et al. 2010; Chiusolo et al. 2010) were sensitive to AmpB-induced DIKI (McDuffie et al. 2016b; Adedeji et al. 2021). In dogs dosed with AmpB, variable increases in urinary ALB and TP were also observed suggestive of an AmpB-induced injury to the proximal nephron. Taken together, these data suggested that AmpB’s effects on the nephrons extend beyond the distal portion.

In a more recent study by Dasgupta et al. (2024), a panel of 6 miRNAs (miRs-210-3p, 423-5p, 143-3p, 130b-3p, 486-5p, 193a-3p) were quantified from the urine samples of cynomolgus monkeys that were dosed with a known, but deidentified nephrotoxicant. The study showed that exosomal-packaged miRNAs had a better correlation than whole urine miRNAs relative to the histopathology scores. Among the 6 miRNAs evaluated by Dasgupta et al. (2024), miR-423-5p was the only miRNA that showed significant increases in rats in our hands. Differences in results between the 2 studies might be due to differences in animal species, assay platforms, nephrotoxicants, and urine-matrix types.

As previously mentioned, there were 35 miRNAs that were significantly differentially expressed across all species in response to the renal injuries in this study. Most of these biomarkers showed significant increases to early renal tubular injury. In mice, the most sensitive miRNAs showed 3- to 29-fold increases on D1 of AmpB dosing correlating with only minimal to mild renal tubular injury in histopathology and comparable to the most sensitive protein biomarker—urine ALB, (↑ 40-fold)—observed in mice (Adedeji et al. 2021). On D3 and D5, miRNA biomarkers showed comparable to higher sensitivity to renal tubular (moderate to marked) relative to the most sensitive protein biomarkers on those days including urine KIM-1 (↑ 31- to 292-fold), ALB (↑ 9- to 45-fold), and NGAL (↑ 17- to 41-fold) (Adedeji et al. 2021).

In rats, there were 9 miRNAs that showed sensitivity to renal injury relative to 5 in mice. Throughout the course of the study, the magnitudes of miRNA increases were marked, ranging from 3- to 164-fold increases with miR-205-5p showing the highest sensitivity on D4. In comparison, as reported by the McDuffie et al. (2016b) in the corresponding protein biomarker study, the most sensitive marker was NGAL with a 14.5-fold increase on D4, indicating that the miRNAs are more sensitive to renal tubular injury than protein biomarkers in rats. Interestingly, there was one miRNA marker, miR-375-3p, that was downregulated with 75% to 91% decreases in expression in response to the renal injury between D4 and D11. This finding is consistent with what was observed in the urine of some patients with diabetic kidney disease due to type 2 diabetes mellitus in which miR-375 was downregulated (Zapała et al. 2023). In contrast, there was another study with cisplatin-induced nephrotoxicity conducted in Dicer^flox/flox^X^cre^Y and P53^flox/flox^X^cre^Y knock-out mice showing upregulation of miR-375 in kidney tissues following cisplatin-induced injury (Hao et al. 2017). In this study, it was shown that miR-375 induction was mediated by P53 and NF-κB following cisplatin administration leading to the repression of HNF-1β to increase tubular cell apoptosis. Hence, miR-375 is believed to be a proapoptotic miRNA. This observation was also supported with the data from the Human MicroRNA Disease Database, showing that miR-375 controls the expression of genes involved in apoptosis (TP53, BCL2, XIAP), cell protection (PRDX1), or protein degradation system (UBE3A) (Zapała et al. 2023). Hence, it is unclear why miR-375 was downregulated in this study with AmpB-induced nephrotoxicity, but the possibility of species-related differences regarding the roles of miR-375 cannot be ruled out. Moreover, miR-375 did not show any change in the AmpB-induced nephrotoxicity in mice in our study. This inconsistency could be due to differences in sample matrices, assay platforms, and mouse strains.

Beagle dogs had the highest number of miRNAs that showed modestly significant upregulations (i.e. 29 miRNAs) in response to the AmpB-induced nephrotoxicity in this study. That said, none of these miRNAs were as sensitive as miR-205-5p (↑67- to 164-fold), miR-31-5p (↑29- to 61-fold), miR-203-3p (↑7- to 37-fold), and miR-423-5p (↑5- to 120-fold) in rats or miR-205-5p (↑29- to 465-fold) and miR-31-5p (↑5- to 270-fold) in mice, as miR-148a and miR-29b were the most sensitive in dogs with 3- to 21-fold and 4- to 16-fold increases, respectively. Most of these miRNAs in dogs were sensitive to early renal tubular injury as their increases correlated with minimal to mild renal tubular injury as observed in the prior publication (Adedeji et al. 2023). However, none of these miRNAs were as sensitive as urine CLU in the AmpB-protein biomarker study (Adedeji et al. 2023), as urine CLU demonstrated 47- to 110-fold increases between D1 and D4 of AmpB dosing. As such, urine CLU might be better than miRNAs for monitoring DIKI in dogs as noted in other studies (Zhou et al. 2014; McDuffie et al. 2016a; Adedeji et al. 2023; Naboulsi et al. 2024).

Taken together, given the location-specificity of the expressions of these miRNAs in the nephron and since a more predominantly distal nephron nephrotoxicant was used in this study, we believe the inclusion of miRNAs for monitoring renal toxicity would be complementary rather than the replacement of the more established urinary protein biomarkers.

Generally, in toxicology studies, large animal toxicology species such as dogs, minipigs, and nonhuman primates have a higher level of biological variability relative to rodents as they usually have different sources of origin compared with rodents. As such, the clinical pathology values obtained from the dosed animals of the large animal toxicology species are often compared with the baseline control values (values obtained prior to dosing) rather than their concurrent control values (the time-matched control values or values obtained from the control group dosed with the vehicle rather than the test article) as in rodents. In this study, the miRNA MFIs of the dogs were compared with their concurrent control MFIs rather than their baseline control MFIs as the MFIs were highly variable in the concurrent control dogs. We reckoned that comparing the dosed animals’ MFIs to their baseline MFIs would have produced fold changes that did not take into considerations the daily variability observed in the concurrent control animals thereby generating fold changes that would be too good to be true. As such, it is our recommendation that for miRNA assessments in dogs, the values should be compared with the concurrent control values rather than the individual animal baseline values.

In conclusion, we have assessed the response of miRNAs to DIKI across 3 animal species commonly used for toxicity studies using a novel FirePlex assay platform. The results showed that miR-205-5p and miR-31-5p were the most consistent miRNA biomarkers across all 3 species and were also the most sensitive markers in mice and rats. In mice, miR-205-5p and miR-31-5p sensitivities were either comparable or slightly better than the urinary protein biomarkers assessed in a previously published report (Adedeji et al. 2021). In rats, these 2 miRNA biomarkers were more sensitive than the most sensitive urine protein biomarkers that have been previously published (McDuffie et al. 2016b). Although, dogs had the highest number of miRNAs that showed response to AmpB-induced kidney injury, none of these miRNAs were as sensitive as urine CLU in its response to nephrotoxic-induced kidney injury in previously published studies (Zhou et al. 2014; McDuffie et al. 2016a; Adedeji et al. 2023; Naboulsi et al. 2024). Taken together, these results will not only add to the body of knowledge of the biomarkers available to monitor DIKI in toxicity studies but could also complement the more established urinary protein biomarkers for monitoring DIKI. For better understanding of miRNA responses to DIKI across multiple preclinical species, more studies with different nephrotoxicants that target distinct nephron segments across multiple preclinical species are needed.

## Data Availability

Supplementary data are available at https://doi.org/10.5061/dryad.c59zw3rk4.
